# Effect of obesity on mood regulation and eating attitudes in mental disorders

**DOI:** 10.1590/1806-9282.20231055

**Published:** 2024-04-22

**Authors:** Hayriye Baykan, Sinan Altunöz, Merve Şahin Can

**Affiliations:** 1 Balikesir University, Faculty of Medicine, Department of Mental Health and Diseases – Balıkesir, Turkey.

**Keywords:** BMI, Eating behaviors, Emotion regulation, Obesity, Mood

## Abstract

**OBJECTIVE:**

The precise relationship between obesity and eating habits, attitudes, and emotion regulation is still ambiguous. The purpose of this study was to investigate possible correlations among body mass index, challenges related to managing emotions, and attitudes toward eating among adult participants with known psychiatric diagnoses.

**METHODS:**

The body mass indices of participants were calculated, and data on eating styles were collected using the Dutch Eating Behavior Questionnaire. The level of difficulty in managing emotions was evaluated using the Difficulties in Emotion Regulation Scale.

**RESULTS:**

The research findings indicated a meaningful positive association. An observation was made between body mass index and results from the Eating Attitude Test-40, as well as the restrained eating subdimension of the Dutch Eating Behavior Questionnaire. Conversely, a meaningful reverse relationship was identified between the scores of the "strategies" subdimension of the Difficulties in Emotion Regulation Scale. No meaningful differences in eating attitudes and emotion regulation were found between non-obese and obese patients.

**CONCLUSION:**

While a partial and meaningful correlation was observed among body mass index, eating attitudes, and emotion regulation difficulties, it is suggested that factors such as patients’ age, disease duration, current body mass index, and the simultaneous presence of depression and anxiety should be considered.

## INTRODUCTION

Body mass index (BMI)≥30 kg/m^2^ is known as obesity and has a multifactorial etiopathogenesis, involving genetic, environmental, metabolic, lifestyle, and behavioral factors^1^. The existence of overweight or obesity elevates the likelihood of numerous diseases, making it a serious global epidemic linked to stroke, heart disease, colorectal cancer, high cholesterol, high blood pressure, gallbladder disease, diabetes mellitus, and increased mortality^2^.

Research indicates a strong bidirectional association among diverse psychiatric disorders and obesity. These conditions can be impacted by factors such as age, gender, and socioeconomic status^3^. Anxiety and mood disorders are widespread among patients looking bariatric surgery for obesity, and binge eating disorder is commonly associated with severe obesity^4^. During life, people are continually unprotected from internal and environmental stimuli that can cause emotional reactions, and these feelings manifest themselves with some cognitive, behavioral, and physiological responses^5^. The moment emotion is experienced, and the presence of emotion regulation comes into question^6^.

Emotion regulation is essential for processing and managing emotions effectively. It involves skills to identify, comprehend, and accept emotions, using adaptive regulation strategies based on individual goals and values^7^.

People may turn to eating as a coping mechanism when faced with negative emotions. Fernandes et al., found varied emotional processing and regulation challenges in obese individuals with and without binge eating disorder. Shriver et al., suggested that in adolescents, lack of emotion regulation predicts future obesity through emotional eating^8,9^.

Our research explored the relationship between BMI, emotion regulation challenges, and eating attitudes in adults with psychiatric diagnoses at a psychiatry clinic. We also investigated the relationship between psychiatric diagnoses, emotion regulation difficulties, and eating attitudes. Our hypothesis is that higher BMI would be linked to more eating attitude disorders and emotion regulation difficulties, which are associated with underlying psychiatric disorders. Addressing these goals will provide information for interventions for individuals with psychiatric disorders and obesity.

## METHODS

Before starting the study, ethical approval (No. 2022/123) was obtained from Balikesir University Health Sciences Non-Interventional Research Ethics Committee on 03.08.2022. Participants were selected from individuals seeking treatment at the Psychiatry Clinic. Inclusion criteria were adults aged 18–68 years with known psychiatric diagnoses. Exclusion criteria were severe cognitive impairment or inability to provide informed consent.

### Procedure

Participants provided information on purpose, procedures, risks, and benefits. Verbal and written consent was obtained, emphasizing voluntary participation and the right to withdraw without repercussions. The size of the study group was determined using the G-Power 3.1 software (α=0.05 and power=80%), resulting in 140 participants. The final sample was 123 adults seeking treatment at the Psychiatry Clinic, with 78.4% females (n=96) and 21.96% males (n=27). The age range was 18–68 years, with an average age of 37.94 (SD=12.09) years.

### Data collection tools

#### General Information Form

Researchers utilized a sociodemographic data form they developed to assess participants’ socio-demographic participant characteristics, including age, gender, and other relevant information.

#### The Difficulties in Emotion Regulation Scale

The Difficulties in Emotion Regulation Scale (DERS) by Gratz and Roemer assessed participants’ emotion regulation challenges. It is a 5-point Likert-type scale (1=almost never to 5=almost always) without a specified cut-off score. Higher scores indicate more severe challenges. DERS has six subdimensions: Awareness (components 2, 6, 8, 10, 17, 34, and 34), Clarity (components 1, 4, 5, 7, and 9), Rejection (components 11, 12, 21, 23, 25, and 29), Strategies (components 15, 16, 22, 28, 30, 31, 35, and 36), Impulse (components 3, 14, 19, 24, 27, and 32), and Goal (components 13, 18, 20, 26, and 33). The internal consistency coefficient of the scale is 0.93, and the subdimensions range from 0.80 to 0.89^10^.

#### Eating Attitude Test

The Eating Attitude Test-40 (EAT-40) is a 6-point Likert-type self-report measure (ranging from "Always" to "Never") with 40 items. Developed by Garner and Garfinkel, it identifies individuals at risk for eating disorders and assesses symptoms. Applicable to ages 11 years and above, specific items have different scoring criteria. Scores≥30 on the EAT-40 indicated "prone to eating behavior disorder" in this study. Savaşır and Erol examined the validity of EAT-40 in Turkey, yielding a reliability coefficient of 0.65, and Cronbach's alpha coefficient was 0.70 for internal consistency^11^.

#### The Dutch Eating Behavior Questionnaire

Van Strien et al., developed a 33-item scale assessing emotional, restrained, and external eating styles which includes 10 items for restrained eating, 13 for affective eating, and 10 for extrinsic eating. Responses range from "1: Never" to "5: Very often," with item 31 scored reversely. Psychometric characteristics were studied in a Turkish university sample. Cronbach's alpha was 0.94 for the entire scale, 0.90 for external eating, 0.91 for restrained eating, and 0.97 for emotional eating subdimensions. The test–retest reliability was 0.72, indicating DEQB's reliability in Turkey^12^.

#### Body Mass Index Measurement

BMI is a common noninvasive measure for obesity assessment. Height was accurately measured after removing shoes and excess clothing. BMI was calculated using the formula BMI=Weight (kg) / Height (m)^2^. Participants were categorized based on BMI values: underweight (<18.50 kg/m^2^), normal weight (18.50–24.99 kg/m^2^), overweight (25.0–29.99 kg/m^2^), and obese (≥30.0 kg/m^2^)^13^.

### Statistical analysis

Study data were analyzed using the SPSS 25.0 software. Descriptive statistics (N, %, $\bar{x}$ , SD, M) summarized data. Normality was assessed with the Kolmogorov-Smirnov test and histograms. The independent-samples t-test compared normally distributed quantitative data. The Mann-Whitney U test analyzed non-normally distributed data. The Spearman correlation coefficient was used for non-normally distributed quantitative variables, and the Pearson correlation coefficient was used for normally distributed variables. The chi-square test compared categorical data. Test selection was based on the nature of the data and research hypotheses. The significance level of p<0.05 and a 95% confidence interval were used for the assessment of results.

## RESULTS

Table 1 shows the sociodemographic data and psychiatric diagnoses of participants [n=123, with 78.04% females (n=96) and 21.96% males (n=27)]. The average age of the participants was 37.94±12.09 years (range: 18–68 years). The average BMI of the participants was 28.98±6.83 kg/m^2^ (range: 15.00–45.50 kg/m^2^). The diagnoses were depressive disorder (51.21%, n=63), anxiety disorder (39.02%, n=48), and obsessive-compulsive disorder (9.75%, n=12).

**Table 1:** Descriptive statistics.

	Female (n=96)	Male (n=27)	Total (n=123)
Gender	78.04%	21.96%	100%
Age (mean±SD) (years)	37.38±11.92	39.96±12.73	37.94±12.09
Body mass index (mean±SD) (kg/m^2^)	28.24±7.33	29.37±4.64	28.49±6.83
Depressive disorder % (n)	36.58 (45)	14.63 (18)	51.21 (63)
Anxiety disorder % (n)	33.33 (41)	5.69 (7)	39.02 (48)
Obsessive-compulsive disorder % (n)	8.13 (10)	1.62 (2)	9.75 (12)

There were 50.40% (n=62) obese (BMI≥30 kg/m^2^) and 49.59% (n=61) nonobese participants. The average ages of the obese and nonobese groups were 44.27±11.11 and 31.51±9.40 years, respectively. A statistically significant relationship was found between EAT-40 scale scores and BMI (r=0.233; p=0.01). Particularly in depressive disorder individuals, EAT-40 scores significantly correlated with BMI (r=0.249; p=0.049).

A noteworthy negative correlation was observed between DERS2 (Strategies) results and BMI (r=-0.184; p=0.041). A statistically significant negative correlation was observed in anxiety disorder patients (r=-0.296; p=0.022), but not in depressive disorder patients (r=-0.043; p=0.740).

A statistically meaningful correlation was found between DEBQ1 (restrained eating) results and BMI (r=0.212; p=0.019). In depressive disorder volunteers, a significant correlation between DEBQ1 (restrained eating) results and BMI (r=0.282, p=0.025) was observed, but not in anxiety disorder patients (r=0.132; p=0.315).

No statistically substantial correlations were found in paired comparisons between obese and nonobese groups using the study scales (Table 2). The average scores obtained from the scales used in the study are presented in Table 3.

**Table 2:** Correlation between the scales utilized in the study and body mass index.

p<0.05*	Body mass index					
	All participants		Depression		Anxiety disorder	
	r	p	r	p	r	p
EAT-40	0.233	0.010	0.249	0.049	0.230	0.078
DEBQ1 (Restrained eating)	0.212	0.019	0.282	0.025	0.132	0.315
DEBQ2 (Emotional eating)	0.059**	0.515	0.063	0.625	0.037	0.776
DEBQ3 (External eating)	0.017	0.849	0.009	0.945	0.095	0.472
DERS1 (Goal)	0.105	0.250	0.210	0.099	-0.021	0.873
DERS2 (Strategies)	-0.184	0.041	-0.043	0.740	-0.296	0.022
DERS3 (Impulse)	0.087	0.341	0.169	0.185	-0.082	0.536
DERS4 (Awareness)	0.026	0.779	-0.059	0.645	0.008	0.954
DERS5 (Clarity)	0.131	0.148	0.153	0.233	0.052	0.694
DERS6 (Nonacceptance)	-0.042	0.641	-0.022	0.863	-0.124	0.344

* Level of statistical significance.

** Spearman correlation coefficient.

**Table 3:** Mean (±SD) scores obtained from the scales used.

Scales	Mean±SD
EAT-40	20.87±10.68
DEBQ1 (Restrained eating)	24.24±8.91
DEBQ3 (External eating)	28.00±8.44
DERS1 (Goal)	14.80±6.18
DERS2 (Strategies)	16.98±6.02
DERS 3 (Impulse)	14.92±6.02
DERS4 (Awareness)	16.42±4.50
DERS5 (Clarity)	20.85±8.41
DERS6 (Nonacceptance)	12.94±4.52
Scale	Med. (min.–max.)
DEBQ2 (Emotional eating)	20 (13–65)

## DISCUSSION

Previous studies in the literature have documented that those people with eating disorders have at least one psychiatric comorbidity before or concurrently with the establishment regarding the identification of an eating disorder and persisted after the eating disorder has been cured^14^. Depression is more prone to occur after the progress of an eating disorder, while anxiety tends to manifest before the progress of an eating disorder^15^. According to this, the hypothesis was formulated that anxiety may be linked to more severe symptoms of the eating disorder^16^. Based on the current literature data, our research found that the average EAT-40 result was below the cutoff point (20.87±10.68). This could explain the absence of significant correlations between BMI, EAT-40, and restrained eating subdimension scores from the DEBQ1 in patients with anxiety disorder.

Anxious mood showed a selective association with eating psychopathology in anorexia nervosa participants compared with other affective temperaments. However, the significant effect of depressive mood on anorexia nervosa psychopathology was also emphasized^17^.

Our research detected a significant association among BMI, eating attitudes, and challenges in emotion regulation. However, unlike the existing literature, no notable distinction was detected between the obese (BMI≥30 kg/m^2^) and non-obese patient groups regarding eating attitudes and emotion regulation difficulties. We acknowledge that our study's clinical sample and cross-sectional approach may introduce confounding factors. Important limitations include environmental influences, disease duration, current BMI, and levels of anxiety and depressive symptoms between the non-obese and obese groups.

Previous research emphasizes the effect of energy intake variations and individual eating behavior differences on obesity. Morbidly obese patients often exhibit problematic eating behaviors. Categorizing eating behaviors into emotional, external, and restrained styles is common and relevant in obesity etiological models^18,19^. Despite variations in the data from different studies, a notable link has been observed between restrained eating behaviors and obesity as well as BMI^20–22^. Our study uncovered a statistically meaningful association among the scores of the restrained eating subdimension in the DEBQ and BMI, which aligns with the findings from previous research.

Difficulties in emotion regulation are significant in individuals with eating disorders, often leading to unhealthy eating habits as a means of coping with negative emotions^23,24^. Many studies have suggested that unhealthy eating habits could be due to efforts to regulate negative emotions. Studies comparing healthy and obese volunteers have shown that obese individuals more frequently face emotion regulation challenges. However, investigations on high BMI individuals without eating disorders are limited^25^. In our study, a clear negative correlation was found between the "Strategies" subdimension results of the DERS2 scale and BMI.

The absence of statistically meaningful distinction between obese (BMI≥30 kg/m^2^) and non-obese groups in eating attitudes and emotion regulation challenges suggests the role of environmental factors, disease duration, age, BMI, and comorbidities (depression and anxiety) in influencing outcomes.

### Significant implications for clinical practice

Clinicians should conduct a thorough assessment of eating attitudes and emotion regulation in patients with psychiatric diagnoses, as these factors significantly relate to BMI. Special attention should be given to restrained eating behavior, which shows a positive correlation with BMI. Comorbidities such as depression and anxiety should be considered when evaluating these relationships. Individualized treatment plans should be developed, considering factors such as age, disease duration, current BMI, and comorbidities. Further research, including healthy volunteer studies, is necessary to better understand the complexities of these correlations. A multidisciplinary approach involving mental health professionals and nutritionists may be beneficial in providing comprehensive care to patients.

### Limitations

Cross-sectional design hampers causal relationships. Longitudinal studies better explore BMI, emotion regulation, and eating attitudes over time. Focusing on clinical sample limits generalizability. Healthy controls would offer a comparative perspective. Relying on self-reports may introduce response bias and miss variable complexity. Future studies need objective assessments and diverse, larger samples to address these limitations.

## CONCLUSION

The research found significant correlations between BMI, eating attitudes, and emotion regulation challenges in adult patients with psychiatric diagnoses at a psychiatry clinic. Higher BMI correlated positively with restrained eating and negatively with emotion regulation strategies. Interventions targeting emotion regulation and maladaptive eating attitudes could benefit individuals with psychiatric diagnoses and high BMI. Future research should explore longitudinal associations, use larger, diverse samples, and include healthy volunteers to understand BMI, emotion regulation, eating attitudes, and psychiatric diagnoses better. The research supports targeted interventions for individuals with psychiatric disorders and obesity.
